# Editorial: Evaluating surgical techniques and perioperative strategies in colorectal cancer treatment

**DOI:** 10.3389/fsurg.2026.1928265

**Published:** 2026-07-28

**Authors:** Gianpiero Gravante, Dragomir Dardanov, Wenpeng Wang, Shengtao Lin

**Affiliations:** 1 Department of General Surgery, Azienda Sanitaria Locale ASL Lecce, Casarano, Italy; 2 Lozenetz Hospital, Sofia, Bulgaria; 3 Tianjin Medical University Cancer Institute and Hospital, Tianjin, China; 4 Fuzhou University, Fuzhou, China

**Keywords:** cancer, colorectal surgery, eras (enhanced recovery after surgery), prehabilitation, staging & surgical approach

Colorectal cancer surgery has moved beyond an era in which progress was measured almost exclusively by the ability to perform a safe resection. Contemporary surgical quality is now defined by a broader and more demanding framework: oncological radicality, preservation of function, prevention of complications, perioperative recovery, pathological accuracy, survivorship, cost-effectiveness, and – above all - the capacity to individualize treatment. This Research Topic captures that transition. The included contributions do not simply describe isolated technical refinements; collectively, they show how colorectal cancer surgery is becoming an integrated pathway in which operative technique, perioperative optimization, risk prediction, pathology, nursing care, reconstruction, and long-term functional recovery are increasingly interconnected. A purely descriptive summary of the articles would underestimate the value of the Research Topic.

Across the 21 contributions, five major areas emerge: oncological quality and staging, technology-enabled surgical precision, prediction and mitigation of perioperative risk, optimization of recovery through ERAS and prehabilitation, and patient-centered survivorship. These areas are not separate, but represent different expressions of the same evolution: from procedure-centered colorectal surgery to personalized colorectal cancer care.

## Oncological quality and staging: from resection to measurable precision

The first thematic area concerns oncological adequacy. Lymph node retrieval, D3 dissection, fluorescence guidance, and pathological processing are presented in the Research Topic not as isolated technical endpoints, but as determinants of staging quality and treatment planning (Ma et al., Biswas et al., Pacilli et al., Wang et al.).

The studies addressing lymph node yield emphasize that the traditional threshold of 12 nodes remains clinically important, but also imperfect. It is affected by patient factors, tumor stage, neoadjuvant therapy, anatomical complexity, surgical dissection, and the methodology of pathological examination (Ma et al., Biswas et al.). Therefore, lymph node count should be interpreted as a multidisciplinary quality indicator rather than as a simple surrogate of surgical performance. This concept is strengthened by the work on fluorescence-guided surgery and right-sided D3 lymphadenectomy (Pacilli et al., Wang et al.). Indocyanine green may support intraoperative lymphatic mapping and perfusion assessment, while a more extended medial boundary for D3 dissection may increase nodal yield. However, the Research Topic also reminds us that greater visualization or wider dissection must ultimately be judged against clinically meaningful outcomes: margin status, recurrence, survival, morbidity, and value. The challenge for future research is not merely to demonstrate that more nodes can be retrieved, but to clarify when additional staging information changes adjuvant treatment, follow-up intensity, or survival.

## Surgical innovation and robotic surgery: selective value rather than universal superiority

A second major theme is the evaluation of surgical innovation. The studies on intracorporeal anastomotic configuration, robotic proctectomy, robotic platform evolution, robotic intersphincteric resection, and robotic versus laparoscopic colorectal surgery converge on a cautious but important conclusion: innovation should be assessed according to indication, anatomy, complexity, learning curve, and patient-centered benefit rather than by technological enthusiasm alone (Fangxin et al., Wang et al., Wang et al., Al-Ihribat et al., Zhou et al.).

The comparison between overlap and pi-shaped ileotransverse anastomosis in laparoscopic right hemicolectomy illustrates this principle well (Fangxin et al.). A reconstruction technique may improve operative efficiency, inflammatory response, bowel recovery, and tolerance of oral intake without necessarily proving superiority for anastomotic leakage, particularly when event rates are low and statistical power is limited. The robotic studies add further nuance (Wang et al., Wang et al., Al-Ihribat et al., Zhou et al.). Robotic surgery may offer advantages in selected rectal cancer scenarios, especially in the low pelvis, where access, visualization, instrument articulation, and precision may affect circumferential margin status, mesorectal integrity, sphincter preservation, and functional outcomes. At the same time, the Research Topic shows that robotics is not automatically superior to laparoscopy in all patients. Longer operative time, increased costs, platform-specific performance, and institutional experience remain critical variables. The evolution from Si to Xi platforms demonstrates that “robotic surgery” is not a fixed exposure; it changes with technology, docking efficiency, team familiarity, and case selection (Wang et al.). Future comparative studies should therefore avoid simplistic robotic-versus-laparoscopic framing and instead identify the patient, tumor, and system-level contexts in which robotic surgery definitely provides real incremental value.

## Risk prediction and complication prevention: making surgical decisions more individualized

The third thematic area is perioperative risk. Several contributions focus on anastomotic fistula, moderate-to-severe complications after primary tumor resection in metastatic colorectal cancer, experimental effects of 5-fluorouracil on anastomotic integrity, parastomal hernia, and complex pelvic reconstruction (Wei et al., Chernyshenko et al., Zou et al., Shang et al., Wubetu et al.). Their common message is that postoperative complications should not be regarded as unpredictable events occurring after the operation; instead, they should be anticipated, modeled, and, where possible, modified before and during surgery.

Prediction models for anastomotic fistula and postoperative complications are particularly relevant in this respect (Wei et al., Zou et al.). Variables such as tumor location and stage, operative duration, albumin level, and patient-related characteristics represent modifiable or actionable domains that may influence nutritional optimization, diversion strategy, operative planning, postoperative surveillance, and informed consent. Similarly, the experimental meta-analysis on 5-fluorouracil and anastomotic healing highlights the biological dimension of anastomotic safety (Chernyshenko et al.). Although animal data cannot directly dictate clinical practice, they help frame anastomotic leakage as a biological and mechanical event influenced by oncological therapy, tissue repair, inflammation, and timing.

The work on parastomal hernia and flap reconstruction further broadens the meaning of surgical risk (Shang et al., Wubetu et al.). For patients requiring abdominoperineal resection, extralevator resection, exenteration, or permanent stoma formation, the relevant postoperative burden extends well beyond anastomotic leakage. Abdominal wall morbidity, stoma complications, perineal wound problems, reconstructive requirements, and length of recovery are central components of outcome quality. These studies reinforce the need for individualized reconstructive planning, appropriate patient selection, and collaboration between colorectal, plastic, radiological, oncological, and nursing teams.

## ERAS and prehabilitation: optimizing the pathway, not only the operation

A fourth area is perioperative optimization. The ERAS and prehabilitation studies demonstrate that recovery after colorectal cancer surgery is determined by the entire perioperative pathway, not only by the technical success of the operation (Prasitvarakul et al., Fabozzi et al., Tai et al.). ERAS implementation in different healthcare contexts was associated with shorter postoperative hospitalization without an evident penalty in readmission, mortality, or major complications (Prasitvarakul et al., Fabozzi et al.). This is clinically important because it confirms that structured recovery pathways can be transferred across institutions and health systems, provided that multidisciplinary adherence is achieved. Prehabilitation extends this concept into the preoperative period (Tai et al.). Inspiratory muscle training, aerobic exercise, nutritional supplementation, and psychological support are not peripheral interventions; they are attempts to improve physiological reserve before surgical stress. The implication is that colorectal cancer surgery should be understood as a continuum beginning at diagnosis. The waiting time before surgery or neoadjuvant treatment should not be passive. It can be used to improve physical function, nutritional status, respiratory performance, and psychological readiness. Prehabilitation and ERAS should be seen as complementary strategies: one prepares the patient for surgery, the other standardizes recovery after it.

## Functional outcomes and survivorship: the endpoint after oncological success

The fifth area is survivorship. Several articles move the focus from perioperative metrics to bowel function, dietary management, psychosocial adaptation, and quality of life after rectal cancer surgery (Zhang et al., Wen et al., Luo et al.). This is one of the most important conceptual shifts in the Research Topic. A technically successful low anterior resection or permanent colostomy can still leave patients with persistent functional impairment, altered body image, psychological distress, and major changes in daily living. The study of low anterior resection syndrome trajectories is especially relevant because it challenges the limitations of single time-point assessment (Zhang et al.). Patients do not all follow the same recovery curve after ileostomy reversal. Some improve steadily, some fluctuate, and others experience severe persistent symptoms. Such trajectory-based analysis may allow earlier identification of patients who require targeted interventions. The scoping review on nurse-led dietary modification complements this by showing that dietary counseling, fiber management, symptom diaries, and individualized advice may form part of structured functional rehabilitation after sphincter-saving surgery (Wen et al.). Finally, the commentary on acceptance and commitment therapy emphasizes that psychosocial outcomes after permanent colostomy are not secondary to surgical care; they are part of surgical care (Luo et al.).

## Advanced disease and multidisciplinary personalization

The Research Topic also extends beyond localized resectable disease. The review on peritoneal metastases and the study on complications after primary tumor resection in metastatic colorectal cancer emphasize the complexity of surgical decision-making in advanced disease (Zou et al., Stańczak et al.). In this setting, surgery must be integrated with systemic therapy, disease biology, symptom burden, expected survival, patient preferences, and institutional expertise. Cytoreductive surgery, HIPEC, PIPAC, and other locoregional approaches require careful selection, specialized pathways, and realistic outcome assessment (Stańczak et al.). The question is not simply whether surgery can be performed, but whether it improves survival, symptoms, treatment sequencing, or quality of life for the specific patient in front of the multidisciplinary team. This is where the overall message of the Research Topic becomes most evident. Personalized care does not mean applying increasingly complex interventions to every patient. It means selecting the right intervention, at the right time, for the right indication, in the right system. It also means recognizing when a less aggressive strategy, stronger prehabilitation, better stoma counseling, diversion, referral to a specialized center, or avoidance of surgery may represent the most patient-centered decision.

## Future directions

Several priorities emerge for future research. First, predictive nomograms and risk models should undergo external validation across different populations, healthcare systems, and surgical volumes before being adopted into clinical pathways. Second, studies of robotic surgery and fluorescence-guided surgery should include cost-effectiveness, learning curve, functional outcomes, and subgroup analyses based on pelvic anatomy, tumor height, obesity, neoadjuvant therapy, and previous surgery. Third, ERAS and prehabilitation research should move from efficacy to implementation, identifying barriers to adherence and strategies for sustained multidisciplinary delivery. Fourth, functional outcomes should be measured longitudinally using validated tools rather than through isolated postoperative assessments. Finally, future colorectal cancer surgery studies should increasingly use composite endpoints that combine oncological adequacy, complication severity, recovery, function, patient-reported outcomes, and value. The methodological challenge is substantial. Many contributions in this Research Topic are retrospective or single-center, and several promising findings require prospective validation. Nevertheless, the value of the Research Topic lies precisely in mapping the current direction of the field. It shows that colorectal cancer surgery is becoming more data-driven, more technologically sophisticated, more multidisciplinary, and more attentive to functional recovery and survivorship.

Overall, the contributions in the Research Topic support a broader definition of colorectal cancer outcomes and treatment success. Disease-free survival and negative margins remain essential, but nowadays they are insufficient. Future trials and registries should incorporate validated functional scores, patient-reported outcome measures, stoma-related quality-of-life instruments, sexual and urinary function, and psychosocial endpoints. Without these measures, the impact of surgical innovation on the lived experience of patients remains only partially understood.

## Conclusions

This Research Topic demonstrates that the frontiers of colorectal cancer surgery are no longer defined by operative technique alone. Surgical innovation remains essential, particularly in minimally invasive and robotic procedures, fluorescence-guided surgery, complex pelvic surgery, and reconstructive strategies. However, the greatest improvement in patient outcomes is likely to come from integration: precise surgery combined with reliable pathology, risk prediction, ERAS, prehabilitation, multidisciplinary planning, functional rehabilitation, and psychosocial support. The future of colorectal cancer surgery is therefore not simply robotic, laparoscopic, or open. It is personalized, measurable, and pathway-based. Technical excellence remains the foundation, but it must be embedded within perioperative optimization and long-term patient-centered care.

